# Ectrotic Treatment of Small Pox

**Published:** 1847-05

**Authors:** 


*Ectrotic treatment of Small Pox, by* Dr. Crawford.

*To the Editors of the Montreal Medical Gazette.*

Gentlemen,—Will you do me the favor to give a place in the Montreal Medical Gazette, to a suggestion which I wish to offer to my professional brethren, in expectation, that, with their co-operation, it will be found capable of conferring a valuable benefit upon the public.

It is briefly, the application of the tincture of Iodine (*from Magendie*) to prevent the unseemly consequences which attend small pox, and further to render the disease milder and less dangerous, by its peculiar antiphlogistic powers.

I have been in the habit of using this application very extensively in a great variety of affections for some years; particularly in acute rheumatism, neuralgia and erysipelas, more especially that of the face; and have reason to speak of it in the highest terms of commendation. Erysipelas having been very prevalent in this city during the last four years, I have had an opportunity of treating a great number of cases, and although many of these appeared in imminent danger, all except one, (that of an old hospital nurse,) terminated favorably, and it is my conviction, that the mortality would have been much greater, had I not used this application. I would by no means exclude the use of constitutional remedies in this disease, which (although it especially shows itself as a peculiar local inflammation) is essentially dependant on a derangement of the general system; I have, however, on almost all occasions, seen such decided benefit result from its use, when perhaps little or nothing else has been done, that I would rather relinquish the use of every other application or remedy, than resign this one. A distinguished medical practitioner of this city, a short time since, admitted to me that he had not until lately done justice to this remedy, and that he now attributes any unsatisfactory results he had experienced on former occasions, to his not having properly and fully carried out its application. Although it is not my object at present, to extend this notice of its use in erysipelas, I must not omit mentioning, that I have, on many occasions, tested (contemporaneously,) the merits of the several local applications recommended in this disease, and I have no hesitation in assigning a superiority to it above all others. Observing this superiority, and at the same time the similarity in the modus operandi, of this application, and that of nitrate of silver, it occurred to me, to make trial of it in small pox; with the view of preventing pitting and scars, for which object the nitrate of silver has been so frequently used.

A severe case of variola confluenta being admitted into the Montreal Hospital, in the end of September last, on the second day of the eruption, which was attended by considerable tumefaction of the face, the forehead and one cheek was painted with the tincture, the immediate effect of which was to cause a good deal of pain, which however subsided in a short time, and appeared in some degree to remove the burning and itching peculiar to the disease; the application of the tincture was repeated daily, with marked good effects, the tumefaction of the face in some degree subsiding, and the pustules becoming flat, as the remedy appeared to abate the violence of this inflammatory action, on the parts to which it had been applied; it was extended over the whole face; a comparative test was therefore not fully instituted; however, the parts most frequently painted formed much thinner scabs than those which had been less so; these crusts fell off sooner, leaving a surface distinguishable by the fewer pits and slighter marks. Although this case was very severe, and terminated fortunately, it was by no means a favorable occasion for experimenting, the eruption having already been two days out, and the inflammation and tumefaction having attained a considerable height, before the opportunity was afforded for using the application; in addition to which, the cautious and sparing manner in which it was used, necessarily limited its effects materially; however, they were sufficiently evident to encourage further trials and warrant its safety.

Shortly after this, a case of variola discreta occurred in the Hospital, accompanied with considerable fever and delirium; the patient said he never had been vaccinated; the eruption was profuse but distinct. The tincture was applied over the whole face daily from the first day, for about five or six days. The pustules went through their regular stages, but did not accumulate, remaining flat; and the face did not swell. The thin crusts on the face fell off at about the end of a week, leaving it free from any pitting. The postules over the rest of the body filled well, and formed thick scabs, which remained several days longer—one of the hands was also painted to show the contrast, and had a very satisfactory result.

The third case was one of variola modificata; in this case the face was at first only partially painted (as was also one hand) to show a contrast; the good effects were soon evident, and the application was then extended over the rest of the face, to prevent any risk of pitting, as the patient was a good-looking young woman; on the parts most frequently painted, the eruption scarcely formed any pus, and the crusts were very thin, and soon fell off, leaving the parts free even from discoloration, rendering them for some time distinguishable from the others.

The last case that I shall notice, is most particularly satisfactory; not only from its issue, but also from its being under the care of Dr. G. W. Campbell of this city, with whom I frequently visited it.—The violence of the febrile symptoms, and extent of the eruption, led Dr. Campbell to suppose, that it would prove a confluent case. He ordered the tincture to be applied over the whole face, and on visiting the patient the next day, was so pleased with the result, that he directed its application to be made daily; the pustules on the face, although they went through their regular stages, remained flat and small; the face remained free from tumefaction, with the exception of one of the eyelids which was slightly puffed. She had no delirium after the application of the tincture; the crusts, which were very slight on the face, fell off early, leaving it free from pitting, while extensive thick and continuous scabs covered the limbs, and principal parts of the body; and which confined her to bed many days after those on the face had fallen off, giving her a great deal of uneasiness and discomfort. Throughout her complaint, she said her face was her only tolerable part, and although the tincture gave her pain for about an hour after its application, it quite removed the variolous pain and itching, and left her so far comfortable during the rest of the day.

Very little constitutional treatment was resorted to in any of these cases; which have been seen by several members of the profession.

I have heard that some of my medical brethren have been following up the above suggestion, and I learn the application has given satisfaction; my object, however, not being for the purpose of recording cases, but rather to offer a hint generally to the profession, that the application may be fully and fairly tested, I have preferred giving my own personal experience on the present occasion.

I believe almost every one will admit the inefficacy of the several applications hitherto recommended, for the above contemplated object, as well as the disagreeable nature of most of them, or the difficulty of their application. The tincture of iodine will be found, I apprehend, not only more efficacious, but also more manageable and endurable by the patient; I am of opinion that the advantages derivable from its use, will in a great measure depend on its employment in the earliest stages of the eruption, and its steady and daily repetition,—by which means the inflammatory action is moderated, and thereby the destruction of the cutis vera, and subcutaneous cellular substance, and consequent pitting prevented; and also from the relief it affords to the itching, preventing the involuntary scratching and tearing, so frequently a cause of great evil; how far it may be judicious to make a more extended application of the remedy over the body, I am not prepared to say; from what I have witnessed, I feel favorably disposed to it.

I shall trespass a moment longer, to notice an observation which has been made to me on one or two occasions, namely, "are we not likely, by an interference with the progress of a specifi: disease, to repel a morbid poison on the system, which nature appears to be endeavoring to throw off?" Without attempting any refutation of this antiquated view of the pathology of the disease, I shall merely notice that the regular progress of the eruption is not interfered with, that the moderating of the inflammatory symptoms, by this application, renders the disease milder, and it is evident that whatever tends to effect this object, without depressing the vital powers, will be the surest means of saving the life of the patient, and of obtaining the other dreaded consequences. * I am, gentlemen, your obedient servant,

JAMES CRAWFORD, M. D.

*Montreal, March* 15, 1844.

